# Therapy-related leukaemias with balanced translocations can arise from pre-existing clonal haematopoiesis

**DOI:** 10.1038/s41375-021-01150-3

**Published:** 2021-02-05

**Authors:** Richard Dillon, Matthew J. Ahearne, Lynn Quek, Nicola Potter, Jelena Jovanovic, Nicola Foot, Mikel Valganon, Sandrine Jayne, Mike Dennis, Kavita Raj, Sudhir Tauro, Martin J. S. Dyer, Nigel Russell, Ellen Solomon, David Grimwade

**Affiliations:** 1 Department of Medical and Molecular Genetics, King’s College, London, UK; 2 Department of Haematology, Guy’s and St Thomas’ Hospitals NHS Trust, London, UK; 3 Cancer Genetics Service, Viapath, Guy’s Hospital, London, UK; 4 The Ernest and Helen Scott Haematological Research Institute, University of Leicester, Leicester, UK; 5 Molecular Haematology Unit, Weatherall Institute of Molecular Medicine, Oxford, UK; 6 Department of Haematology, King’s College, London, UK; 7 Department of Haematology, The Christie Hospital, Manchester, UK; 8 Department of Haematology, Ninewells Hospital and Medical School, Dundee, UK

**Keywords:** Oncogenesis, Acute myeloid leukaemia

## To the Editor:

Therapy-related leukaemia is a life-threatening complication of cancer treatment. The prognosis is poor and incidence is increasing, mainly due to the increasing survival from primary cancers, leading to an emerging major healthcare problem [1].

Currently two groups of tMN are recognised: the first, accounting for ~80% of cases, is characterised by exposure to alkylating agents, a latency period of 5–6 years, a complex or monosomal karyotype and/or mutations in *TP53* [2]. These mutations have been detected in samples pre-dating therapy exposure [3] and presence of clonal haematopoiesis (CH) prior to cancer treatment is associated with a markedly increased risk of tMN with estimated hazard ratios of 5.8–13.7 [4, 5], indicating that cancer therapy may drive the expansion of pre-existing mutated clones, eventually leading to malignant transformation.

The second group of tMN accounting for ~20% of cases is characterised by exposure to topoisomerase II (topo2) targeting agents, a latency period of 1–2 years and presence of recurrent fusion genes identical to those seen in de novo AML [6, 7]. In these cases, there is evidence that drugs targeting topo2 directly initiate chromosome rearrangements: in the presence of mitoxantrone or etoposide, topo2 cleaves the *PML* and *RARA* loci at the precise base pair positions observed to be fusion breakpoint junctions in patients who develop therapy-related acute promyelocytic leukaemia (tAPL) after exposure to these agents [8, 9].

Fusion-gene associated chromosome translocations are widely regarded as primary leukaemia-initiating events in AML. Genome-wide studies show striking exclusivity between fusion genes and other mutations shown to be leukaemia-initiating events. For example, mutations in *DNMT3A* are frequent in AML and are associated with a preleukaemic state [10–12]. These mutations either do not occur or are rare in leukaemias with fusion genes [11–13]. Thus, two distinct processes that lead to the generation of tMN appear to mirror two separate mechanisms of de novo leukaemogenesis, with two distinct types of primary leukaemia-initiating event (fusion genes and preleukaemic mutations, respectively).

The relationship between CH and therapy-related leukaemias with recurrent chromosome translocations has not previously been investigated. Here, we focussed on a molecularly defined group of patients with *PML-RARA*^+^ tAPL and present evidence that in these patients, in contrast to de novo APL, chromosome translocations may be secondary events, arising on a background of pre-existing CH.

We first performed whole-exome sequencing of 55 cases of de novo (dnAPL) and 13 cases of therapy-related APL (tAPL, exposure details shown in Supplementary Table 1). Information regarding library preparation, sequencing and analysis is provided in the Supplementary data. We identified a mean of 17.1 and 15.3 somatic mutations per exome, respectively (*p* = 0.59). There were no significant differences in the numbers of non-synonymous single nucleotide variants (SNV, mean 8.8 vs. 8.2, *p* = 0.69) synonymous SNV (7.0 vs. 5.8, *p* = 0.49) or InDel mutations (1.3 vs. 1.4, *p* = 0.72).

To identify recurrent APL-associated mutations, we combined this data set with three other whole-exome sequencing studies of APL [12–14] for a total of 164 sequenced cases, and identified 70 recurrently mutated genes (i.e. mutated in two or more cases of APL). We identified a mean of 2.0 and 2.2 recurrent mutations per patient for dnAPL and tAPL, respectively (median 2 range 0–6 and median 2 range 0–5, *p* = 0.74, Supplementary Fig. 1). There was no clear difference in the pattern of mutated genes. There were three genes that were mutated in tAPL but not dnAPL (*AP3D1*, *DNMT3A* and *GPR158*, Fisher’s exact test, *p* = 0.035).

**Fig. 1:**
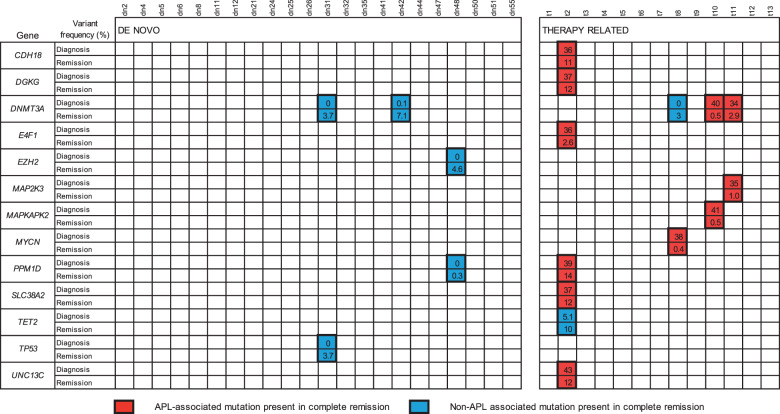
Presence of somatic mutations during molecular complete remission. Mutations identified by error-corrected next-generation sequencing performed on bone marrow samples taken during molecular complete remission after treatment for de novo (*n* = 22) and therapy-related APL are shown. All remission samples tested negative for *PML-RARA* fusion transcripts at a sensitivity of at least 1:10^4^. Variant allele frequencies (%) at diagnosis and remission are shown. Mutations present at a frequency of <1% at APL diagnosis (i.e. APL-unrelated mutations) are highlighted in blue. Mutations which were present with a frequency of >30% at APL diagnosis (i.e. APL-associated mutations) are highlighted in red.

**Fig. 2:**
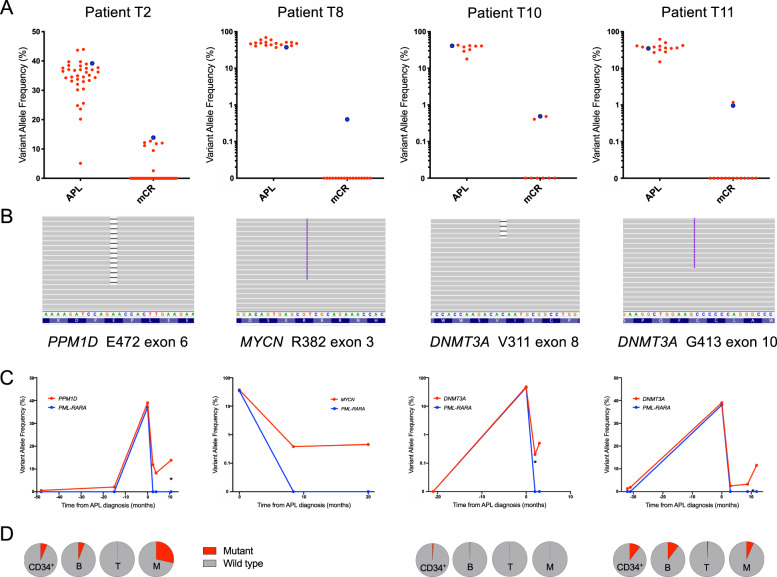
APL-associated mutations persist in molecular complete remission, can occur prior to chemotherapy exposure and affect multiple haematopoietic lineages. **A** Variant allele frequencies (VAF) of somatic mutations detected at diagnosis of therapy-related APL are plotted for both the APL diagnosis (APL) and molecular complete remission (mCR) time points for four patients in whom these remained detectable. Predicted driver (PD) mutations are highlighted in blue. **B** Browser views showing the presence of PD mutations in mCR samples. **C** Change in VAF of APL-associated mutations (red line) and *PML-RARA* fusion transcript levels (blue line) over time, with date of tAPL diagnosis plotted as time 0. **D** VAF of APL-associated PD mutations in sorted cell populations obtained from samples taken in molecular complete remission (the relevant sample is marked with an asterisk in **C**). CD34+ = CD34+ stem and progenitor cells, B = CD19+ B lymphocytes, T = CD3+ T-lymphocytes, M = CD33+ myeloid cells.

We next analysed samples taken in molecular complete remission (mCR, defined as the absence of *PML-RARA* or *RARA-PML* fusion transcripts at a sensitivity of at least 1:10^4^) from all 13 tAPL patients and a subset of 22 dnAPL patients who were matched in age and other clinical characteristics (Supplementary Table 2). Targeted, error-corrected next-generation sequencing (EC-NGS) was performed using the HaloPlexHS system (Agilent Technologies, Santa Clara, California) using probes designed to capture all somatic mutations detected at diagnosis (i.e. APL-associated mutations). The mean depth of sequencing was 27,111× with 97 and 84% of bases covered at >1000× and >10,000×, respectively. In the tAPL group, we identified persistence of a median of 2.5 APL-associated mutations in mCR (range 1–8) in four patients, with a median variant allele frequency of 6% (range 0.4–13.9%, Fig. 1a). Hereafter we refer to these as persistent APL-associated mutations. One potential driver mutation was present in each case, these were *DNMT3A* (two patients) *PPM1D* and *MYCN* (Fig. 2a, b). We did not find the persistence of APL-associated mutations in any patient with dnAPL (Fisher’s exact test, *p* = 0.01). We did not observe an association between persistence of APL-associated mutations and patient age, type of primary cancer, type of treatment received or latency period, however this small cohort provided limited power to detect such associations.

We then performed EC-NGS using a separate HaloPlexHS library designed to capture the entire coding region of all genes identified as bearing persistent mutations in mCR, together with those of other genes previously associated with CH (a total of 87 genes, listed in Supplementary Table 3). Mean sequencing depth was 3378× with 98 and 85% of bases covered at >100× and >1000×, respectively. This method identified APL-unrelated mutations (i.e. mutations not present in the major clone at diagnosis) in both the tAPL and dnAPL groups (3/21 dnAPL and 2/13 tAPL, *p* = 1.0, Fig. 1). These mutations either emerged or increased in frequency after treatment and were mainly in genes previously linked with CH, consistent with previously described therapy emergent or rising clones [15].

In order to determine whether persistent APL-associated mutations were present prior to cancer therapy, tumour biopsies were obtained from time of primary cancer diagnosis for three patients (UPN T2, T10 and T11) and analysed by EC-NGS. Using a VAF cut-off at 0.1% we identified APL-associated mutations in four samples from two patients at a median VAF of 0.8% (range 0.6–2.1%, Fig. 2c). The mutations were *PPM1D* (exon 6, 1 bp deletion) in malignant lymph node samples obtained 48 and 15 months prior to APL diagnosis from patient T2, and *DNMT3A* (exon 10, 1 bp insertion) in breast and lymph node samples both obtained 32 months prior to APL diagnosis from patient T11. For patient T10 the relevant mutation was not identified.

To identify the cell lineage(s) harbouring persistent APL-associated mutations, we obtained cryopreserved mononuclear cell preparations from bone marrow samples taken during mCR from the same three patients. Flow sorted T and B lymphocytes, monocytes, granulocytes and CD34+ progenitors were analysed using amplicon-NGS (further details are provided in the Supplementary data). In each case, the predicted driver mutation was present in the haematopoietic stem and progenitor cell containing CD34+ fraction at a median VAF of 3.2% (range 0.5–5.2%) and was present in both the myeloid and lymphoid lineages, consistent with origin in a multipotent haematopoietic stem or progenitor cell (Fig. 2d).

Finally, to establish whether persistent APL-associated mutations were present in functional haematopoietic stem cells, we injected cryopreserved mononuclear cells taken at diagnosis of tAPL from patients T2 and T8 into irradiated NSG mice. A total of 1 × 10^6^ cells were injected into three mice for each patient. We detected human engraftment in 1/3 recipient mice at 18 and 22 weeks from injection for patient T2 and T8, respectively. After sacrifice, flow cytometry analysis demonstrated human myeloid and B-lymphoid engraftment in both mice (Supplementary Fig. 2). Sorted human CD45+ cells tested negative for *PML-RARA* and *RARA-PML* fusion transcripts by RT-qPCR and were also negative for the fusion by fluorescence in-situ hybridisation. DNA extracted from this population was analysed by amplicon-NGS and identified the tAPL-associated predicted driver mutation in each case (T2 *PPM1D* exon 6, 1 bp del, VAF 2.9%; T8 *MYCN* exon 3, 6 bp ins, VAF 1.4%). Interestingly, the observed allele frequencies indicated that the clone harbouring the somatic mutation contributed a minor component to the human engraftment in each case, suggesting that the mutation did not provide a significant selective advantage in this situation.

Taken together these results indicate that in therapy-related but not de novo APL, the *PML-RARA* fusion may be a secondary event, occurring on a background of preleukaemic CH. It is intriguing that this phenomenon was only observed in tAPL. We cannot exclude that this mechanism may rarely underlie de novo APL and speculate that this observation may be accounted for by differences in prevalence, clone size and mutational spectrum of CH between patients exposed to chemotherapy and individuals with no history of cancer.

These findings contribute to the current understanding of the relationship between CH and leukaemia and challenge the previous assumption that fusion genes are always primary transforming events. It will be of interest to establish the role of CH in other types of tMN with balanced translocations, particularly for patients with *KMT2A* rearrangements, where co-operating mutations are not always necessary. Finally, the high prevalence of CH after treatment for tAPL (observed in 4/13 or 31% of patients studied, with the presence of multiple independent clones in 2/13) also has important implications for treatment of tAPL, a condition where chemotherapy-free treatment in the form of arsenic trioxide and ATRA is already available.

## Supplementary information

Data supplement

## References

[1] Guru Murthy GS, Hamadani M, Dhakal B, Hari P, Atallah E (2018). Incidence and survival of therapy related myeloid neoplasm in United States. Leuk Res..

[2] Smith SM, Le Beau MM, Huo D, Karrison T, Sobecks RM, Anastasi J (2003). Clinical-cytogenetic associations in 306 patients with therapy-related myelodysplasia and myeloid leukemia: the University of Chicago series. Blood..

[3] Wong TN, Ramsingh G, Young AL, Miller CA, Touma W, Welch JS (2015). Role of TP53 mutations in the origin and evolution of therapy-related acute myeloid leukaemia. Nature..

[4] Takahashi K, Wang F, Kantarjian H, Doss D, Khanna K, Thompson E (2017). Preleukaemic clonal haemopoiesis and risk of therapy-related myeloid neoplasms: a case-control study. Lancet Oncol.

[5] Gillis NK, Ball M, Zhang Q, Ma Z, Zhao Y, Yoder SJ (2017). Clonal haemopoiesis and therapy-related myeloid malignancies in elderly patients: a proof-of-concept, case-control study. Lancet Oncol.

[6] Pedersen-Bjergaard J, Andersen MK, Johansson B (1998). Balanced chromosome aberrations in leukemias following chemotherapy with DNA-topoisomerase II inhibitors. J Clin Oncol.

[7] Andersen MK, Larson RA, Mauritzson N, Schnittger S, Jhanwar SC, Pedersen-Bjergaard J (2002). Balanced chromosome abnormalities inv(16) and t(15;17) in therapy-related myelodysplastic syndromes and acute leukemia: report from an international workshop. Genes Chromosomes Cancer.

[8] Mistry AR, Felix CA, Whitmarsh RJ, Mason A, Reiter A, Cassinat B (2005). DNA topoisomerase II in therapy-related acute promyelocytic leukemia. N. Engl J Med.

[9] Mays AN, Osheroff N, Xiao Y, Wiemels JL, Felix CA, Byl JA (2010). Evidence for direct involvement of epirubicin in the formation of chromosomal translocations in t(15;17) therapy-related acute promyelocytic leukemia. Blood..

[10] Shlush LI, Zandi S, Mitchell A, Chen WC, Brandwein JM, Gupta V (2014). Identification of pre-leukaemic haematopoietic stem cells in acute leukaemia. Nature.

[11] Papaemmanuil E, Gerstung M, Bullinger L, Gaidzik VI, Paschka P, Roberts ND (2016). Genomic classification and prognosis in acute myeloid leukemia. N. Engl J Med.

[12] Cancer Genome Atlas Research N, Ley TJ, Miller C, Ding L, Raphael BJ, Mungall AJ (2013). Genomic and epigenomic landscapes of adult de novo acute myeloid leukemia. N. Engl J Med.

[13] Madan V, Shyamsunder P, Han L, Mayakonda A, Nagata Y, Sundaresan J (2016). Comprehensive mutational analysis of primary and relapse acute promyelocytic leukemia. Leukemia.

[14] Lehmann-Che J, Bally C, Letouze E, Berthier C, Yuan H, Jollivet F (2018). Dual origin of relapses in retinoic-acid resistant acute promyelocytic leukemia. Nat Commun..

[15] Wong TN, Miller CA, Klco JM, Petti A, Demeter R, Helton NM (2016). Rapid expansion of preexisting nonleukemic hematopoietic clones frequently follows induction therapy for de novo AML. Blood..

