# Supplementary material for: Therapy-related leukaemias with balanced translocations can arise from pre-existing clonal haematopoiesis
Source: Leukemia. 2021 Feb 5;35(8):2407–11. doi: 10.1038/s41375-021-01150-3 (PMC8324469; doi:10.1038/s41375-021-01150-3)
Supplement: Supplementary file 1 — Data supplement [file 41375_2021_1150_MOESM1_ESM.pdf]

Data supplement

Therapy-related leukaemias with balanced translocations may arise from pre-existing clonal haematopoiesis.

Richard Dillon*[1,2,3], Matthew Ahearne[4], Lynn Quek[5,6], Nicola Potter[1], Jelena Jovanovic[1], Nicola Foot[3], Mikel Valganon[3], Sandrine Jayne[4], Mike Dennis[7], Kavita Raj[2], Sudhir Tauro[8], Martin Dyer[4], Nigel Russell[2], Ellen Solomon[1] and David Grimwade[1]

1) Department of Medical and Molecular Genetics, King's College, London, UK
2) Department of Haematology, Guy's and St Thomas' Hospitals NHS Trust, London, UK
3) Cancer Genetics Service, Viapath, Guy's Hospital, London, UK
4) The Ernest and Helen Scott Haematological Research Institute, University of Leicester, UK
5) Molecular Haematology Unit, Weatherall Institute of Molecular Medicine, Oxford, UK
6) Department of Haematology, King's College, London, UK
7) Department of Haematology, The Christie Hospital, Manchester, UK
8) Department of Haematology, Ninewells Hospital and Medical School, Dundee, UK

*Correspondence:

Dr Richard Dillon, Cancer Genetics Lab, Department of Medical and Molecular Genetics, Floor 8, Tower Wing, Guy's Hospital, London SE1 9RT, UK

Tel +44 207 188 2579

Email richard.dillon@kcl.ac.uk

Contents

Contents	page
Supplementary methods	3
Supplementary figures	5
Supplementary tables	7
Supplementary references	10

Supplementary methods

Patients and samples
All samples were taken from patients for the purpose of molecular disease monitoring. Patients with *de-novo* APL were enrolled in the UK NCRI AML17 study (ISRCTN55675535) [16] and all received treatment with the AIDA protocol and achieved molecular complete remission. Patients with therapy-related APL were treated with either the AIDA protocol or all-trans retinoic acid (ATRA) and arsenic trioxide (ATO) according to the AML17 schedule. Patients provided written informed consent for the use of excess diagnostic material for this study, which was approved by the London Westminster Research Ethics Committee (reference number 06/Q0702/140).

Whole-exome sequencing
Genomic DNA libraries prepared using the SureSelectXT Human All Exon V6 (Agilent Technologies, Santa Clara, CA) target enrichment system were sequenced on the Illumina HiSeq 2500 platform (Illumina, San Diego, CA) with 100bp paired-end reads. Reads were aligned to the reference human genome (hg19, b37) using novoalign (Novocraft Technologies, Selangor, Malaysia). Alignments underwent quality control and filtering as previously described [17]. In each sample, >87% of the GENCODE-defined coding bases of the exome was represented by at least 20 reads. Pairwise variant calling was performed using SAMtools and filtered using VarScan2. Only variant sites with coverage of ≥20 reads in both leukemic and remission samples were considered. Alleles present in ≥20% of reads in leukemic samples and <20% in remission samples were identified and annotated.

Targeted deep sequencing
A custom capture panel consisting all somatic variants identified by whole exome sequencing together with the coding regions of genes previously associated with clonal haematopoiesis (see supplementary table 3) was constructed using the HaloPlexHS system (Agilent). Sequencing libraries were constructed using the Agilent Bravo liquid handling system and sequenced using on a HiSeq 2500 instrument. Alignment and generation of consensus read families was performed using SureCall software (Agilent).

Amplicon sequencing
Sequencing libraries were generated using a two step PCR approach. In the first step, target specific primers incorporating adapter sequences were used to amplify the locus of interest. In the second step, a second PCR was used to incorporate 5' and 3' index and adapter sequences. Libraries were sequenced on a HiSeq 2500 instrument and reads were aligned to the reference genome using Novoalign as described above.

Reverse Transcription- quantitative Polymerase Chain Reaction (RT-qPCR)
RNA was isolated from cell samples of interest using Trizol reagent (Life Technologies, Inchinnan, UK) and cDNA was synthesised using the SuperScript3 reverse transcription kit (Life Technologies). qPCR was performed in triplicate using primer sets and conditions according to the Europe Against Cancer Programme using an ABI 7900 instrument (Life Technologies) and *ABL* was amplified in parallel as a control for RNA quality and quantity. Criteria for reporting positivity proposed by Gabert et al [18] were adopted.

Fluorescence *in-situ* hybridisation (FISH)
Cytospin preparations were fixed in methanol and hybridised with a *PML/RARA* dual fusion FISH probe (Cytocell, Cambridge, UK) for subsequent analysis using fluorescent microscopy.

Cell sorting
Cell suspensions were labelled with the following antibodies: CD34 PE/CF594 (Becton Dickinson, BD, Oxford, UK), CD45 FITC (BioLegend, London, UK), CD14 APC (BioLegend), CD3 PE (BioLegend) CD19 PE/Cy7 (BioLegend). Hoechst 33258 was used as a viability marker. Cells were sorted using a FACSAriaII cytometer (BD). A second sort was used to ensure >99% purity.

Xenografts
NOD/SCIDIL2Rg^{-} (NSG) mice between 10-14 weeks of age were irradiated (2Gy) and treated with human immunoglobulin 10mg per gram of body weight by intraperitoneal injection (Privigen, CSL Behring, Haywards Heath, UK). The following day, 10^6 human cells were injected into the tail vein. Mice received ciprofloxacin in their drinking water for four weeks post-transplant. Mice were assessed for engraftment at 12 weeks and then at monthly intervals. Assessment was by bone marrow aspiration from the tibia under general anaesthetic. Aspirates were stained with the following antibodies: human CD45 APC-Cy7, human CD19 APC, human CD33 PE, human CD 3 FITC (all from BioLegend) and analysed using a BD Fortessa flow cytometer. Where engraftment was detected, mice were sacrificed and the bones dissected and crushed. Cell extracts were stained with the same antibody cocktail and human CD45+ cells were sorted using the FACSAriaII. Double sorting was used to ensure >99% purity. Sorted cells were separated DNA and RNA extraction and cytospins were prepared for FISH.

Supplementary figure 1. Somatic mutational profile of de-novo (n=55) and therapy-related (n=13) APL. BCR = breakpoint cluster region (BCR1 *PML* intron 6, BCR2 *PML* exon 6, *BCR3* PML intron 3). Genes which were only found to be mutated in therapy-related APL are shown in bold/underlined.

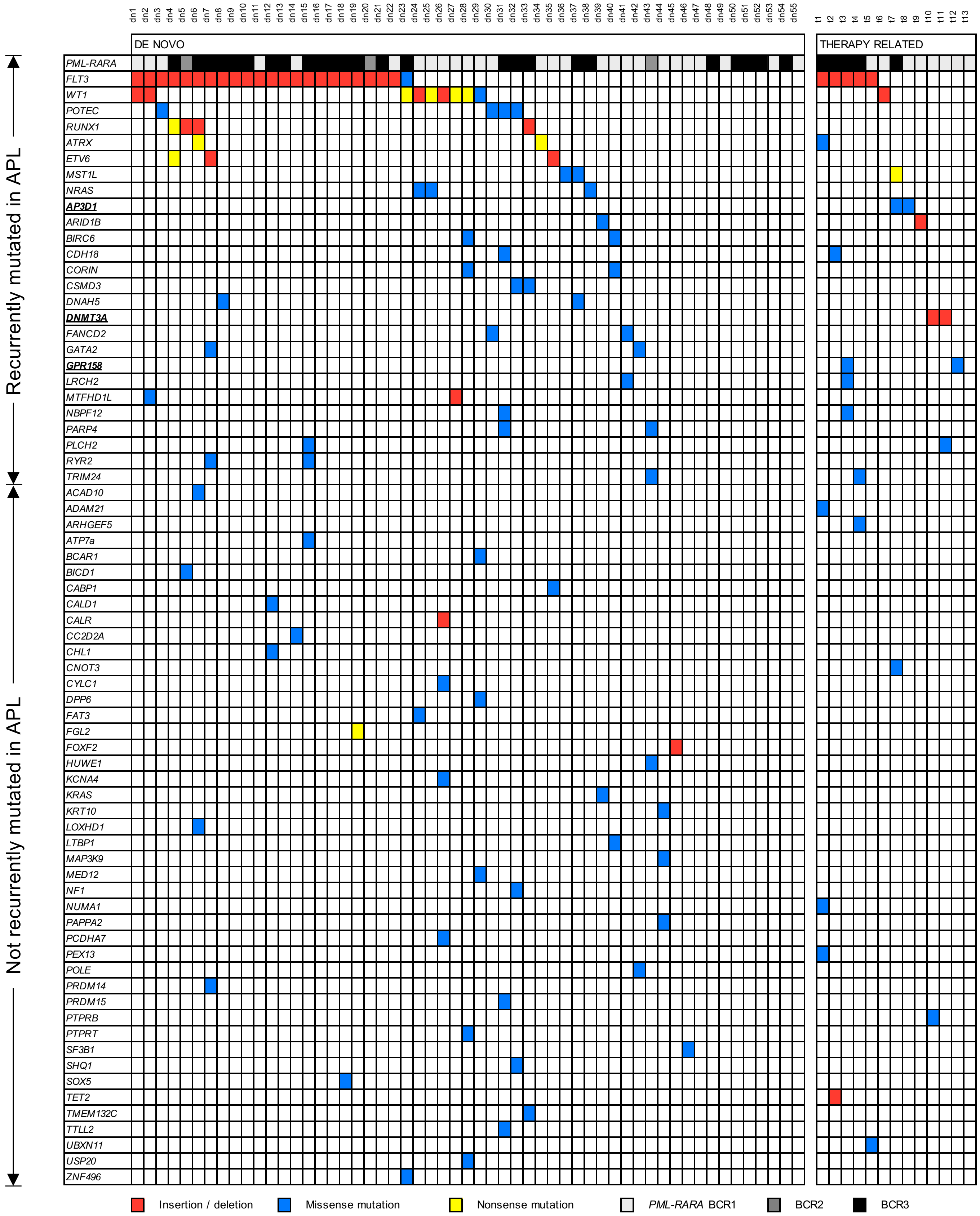

Supplementary figure 2. Multilineage engraftment of tAPL samples in immunodeficient mice. (A) Two samples taken at diagnosis of tAPL were injected in to three irradiated NSG mice each. (B) Human cell engraftment was demonstrated by flow cytometry performed on extracted bone marrow. (C) Engrafting human CD45+ cells consisted of both CD33+ myeloid and CD19+ B-lymphoid cells. (D) Amplicon sequencing on sorted human CD45+ cells demonstrated the presence of the same predicted driver mutations which persisted in molecular complete remission samples from each patient.

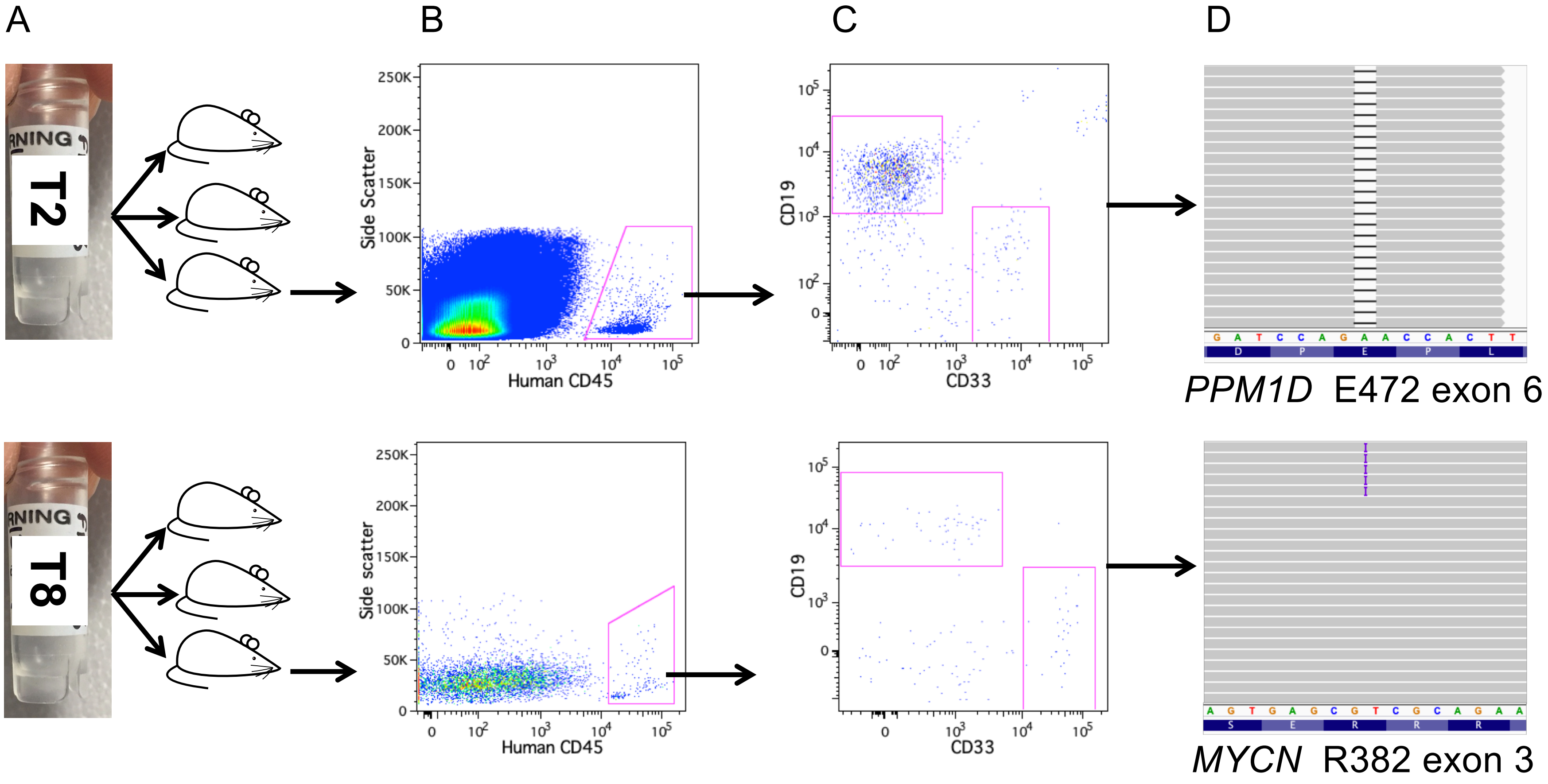

Supplementary table 1. Clinical details and exposure history of patients with therapy-related acute promyelocytic leukaemia.

Case	Age (at APL)	Sex	WBC ($x10^9/L$)	BCR (1/2/3)	Latency (m)	Primary cancer	Chemotherapy	Radiotherapy
T1	63	F	2.9	3	37	Breast	Fluorouracil, Epirubicin, Cyclophosphamide, Docetaxel	No
T2	70	M	26.00	3.00	48	Non-Hodgkin’s lymphoma	Cyclophosphamide, Doxorubicin, Vincristine, Etoposide, Mitoxantrone, Bleomycin, Gemcitabine, Cisplatin	No
T3	64	M	16.00	3.00	47	Colorectal	Oxaliplatin, Fluorouracil	No
T4	30	F	22.00	3.00	37	Hodgkin's lymphoma	Doxorubicin, Bleomycin, Vinblastine, Dacarbazine	No
T5	55	M	0.70	1.00	16	Prostate	None	Yes
T6	47	F	10.00	1.00	65	Breast	Epirubicin	Yes
T7	28	M	0.64	3.00	38	Testicular	Bleomycin, Etoposide, Cisplatin	No
T8	78	F	0.55	1.00	33	Breast	Cyclophosphamide, Docetaxel	Yes
T9	35	F	0.90	1.00	62	Colorectal	Capecitabine, Oxaliplatin	Yes
T10	41	M	0.80	1.00	22	Hodgkin's lymphoma	Doxorubicin, Bleomycin, Vinblastine, Dacarbazine	Yes
T11	53	F	1.10	1.00	32	Breast	Docetaxel, Epirubicin, Cyclophosphamide,	Yes
T12	25	F	2.00	1.00	22	Pituitary	None	Yes
T13	66	F	0.60	1.00	62	Colorectal	Oxaliplatin, Fluorouracil, Capecitabine	Yes

Supplementary table 2. Clinical and molecular details of patients with *de-novo* or therapy-related APL who were selected for targeted deep sequencing of diagnosis and molecular complete remission samples.

	**De-Novo APL (n=22)**	**Therapy-Related APL (n=13)**	**p**
Median age (y)<br>Range	51<br>31-79	53<br>25-78	0.77
Male<br>Female	5 (38%)<br>8 (62%)	12 (55%)<br>10 (45%)	0.49
Median white blood cell count at presentation ($x10^9$/L), range	3.2<br>(0.5-40)	0.9<br>(0.6-22)	0.22
FLT3 ITD positive<br>negative	5 (38%)<br>8 (62%)	9 (41%)<br>13 (59%)	1.00
BCR1<br>BCR3	10 (45%)<br>10 (45%)	7 (54%)<br>6 (46%)	1.00

Supplementary table 3. List of genes included in custom capture panel used to identify clonal haematopoiesis. The entire coding region of all listed genes was included.

ADCY5	*APEH*	*ASXL1*
ASXL2	*ATM*	*ATRX*
AXL	*BCL11B*	*BCOR*
BCORL1	*BRAF*	*BRCC3*
BRD9	*CALR*	*CBL*
CDK13	*CDKN2A*	*CEP41*
CREBBP	*CSF3R*	*CUX1*
DACT1	*DDX3X*	*DIDO1*
DNMT3A	*EZH2*	*FAM46C*
FCN2	*GATA2*	*GNAS*
GNB1	*GPR158*	*GPRC6A*
GUCY1A2	*HDAC4*	*IDH1*
IDH2	*JAK2*	*KDM6A*
KIT	*KMT2D*	*KRAS*
LUC7L2	*MBD1*	*MECOM*
MPL	*MYCN*	*MYD88*
MYL6	*MYLK*	*NOTCH1*
NOTCH3	*NRAS*	*PALLD*
PHF6	*PPM1D*	*PRDM1*
PRKCG	*PRKDC*	*PRRT3*
PTPN11	*PTPRK*	*RAD21*
RICTOR	*RUNX1*	*SETBP1*
SETD2	*SETDB1*	*SF1*
SF3B1	*SH2B3*	*SMARCC2*
SNX25	*SOS1*	*SPATA31E1*
SRSF2	*STAG2*	*STAT3*
TELO2	*TET2*	*TNFAIP3*
TNFRSF14	*TP53*	*U2AF1*
WT1	*ZNF318*	*ZRSR2*

Supplementary references

16. Burnett AK, Russell NH, Hills RK, Bowen D, Kell J, Knapper S, et al. Arsenic trioxide and all-trans retinoic acid treatment for acute promyelocytic leukaemia in all risk groups (AML17): results of a randomised, controlled, phase 3 trial. Lancet Oncol. 2015;16(13):1295-305.

17. Ostergaard P, Simpson MA, Connell FC, Steward CG, Brice G, Woollard WJ, et al. Mutations in GATA2 cause primary lymphedema associated with a predisposition to acute myeloid leukemia (Emberger syndrome). Nat Genet. 2011;43(10):929-31.

18. Gabert J, Beillard E, van der Velden VH, Bi W, Grimwade D, Pallisgaard N, et al. Standardization and quality control studies of 'real-time' quantitative reverse transcriptase polymerase chain reaction of fusion gene transcripts for residual disease detection in leukemia - a Europe Against Cancer program. Leukemia. 2003;17(12):2318-57.
